# Antimüllerian Hormone Levels of Infants with Premature Thelarche

**DOI:** 10.4274/jcrpe.galenos.2019.2018.0293

**Published:** 2019-09-03

**Authors:** Nursel Muratoğlu Şahin, Elvan Bayramoğlu, Hatice Nursun Özcan, Erdal Kurnaz, Melikşah Keskin, Şenay Savaş-Erdeve, Semra Çetinkaya, Zehra Aycan

**Affiliations:** 1 Pediatric Health and Disease Training and Research Hospital, Dr. Sami Ulus Obstetrics and Gynecology, Clinic of Pediatric Endocrinology, Ankara, Turkey; 2 Pediatric Health and Disease Training and Research Hospital, Dr. Sami Ulus Obstetrics and Gynecology, Clinic of Pediatric Radiology, Ankara, Turkey

**Keywords:** AMH, premature thelarche, infancy, mini-puberty

## Abstract

**Objective:**

Antimüllerian hormone (AMH) concentrations in mini puberty are higher than those reported for the prepubertal period. In this study we investigated AMH concentrations in infants with premature thelarche (PT). A healthy control group was used for comparison.

**Methods:**

Forty five female infants with PT, aged between one and three years and a control group consisting of 37 healthy girls in the same age range were included in the study. Bone age, pelvic ultrasonography, and concentrations of luteinizing hormone, follicle-stimulating hormone (FSH), estradiol and AMH of the patient group were evaluated. Only serum AMH concentration of the control group was evaluated.

**Results:**

Median (range) serum AMH concentrations in the subjects were 1.66 ng/mL (11.85 pmol/L) [0.15-6.32 ng/mL (1.07-45.12 pmol/L)] and were significantly lower (p=0.025) than for the control group; 1.96 ng/mL (13.99 pmol/L) [0.60-8.49 ng/mL (4.28-60.64 pmol/L)]. AMH and FSH were negatively correlated (r=-0.360, p=0.015) in infants with PT. There was no correlation between AMH and uterine size, uterine volume, endometrial thickness, fundocervical ratio, ovarian size or volume, follicle size and follicle number.

**Conclusion:**

This is the first study that investigates AMH concentrations in infants with PT. The low AMH levels in these infants and the negative correlation between AMH and FSH suggests that AMH may play a role in suppressing pubertal findings during infancy and that decreased AMH may cause PT in infancy.

**What is already known on this topic?**

Antimüllerian hormone (AMH) levels during mini puberty are higher than those of the prepubertal period. AMH inhibits both initial follicle recruitment and follicle-stimulating hormone (FSH)-dependent follicle growth. Therefore the rising levels of AMH during mini-puberty may be an ovarian response to prevent FSH-induced follicle growth. It has been proposed that premature thelarche (PT) in infants results from transient, partial activation of the hypothalamic-pituitary-ovarian axis with excessive secretion of FSH.

**What this study adds?**

This is the first study to investigate AMH concentrations in infants with PT. It was found that AMH concentrations in infants with PT were lower when compared to healthy controls and a negative correlation between AMH and FSH was identified. It was concluded that a decreased level of AMH may cause PT in infants.

## Introduction

Mini-puberty of infancy refers to the transient activation of the hypothalamic-pituitary-gonadal (HPG) axis during the first few months of life. The follicle-stimulating hormone (FSH) concentration in girls decreases at delivery and increases again with activation of the HPG axis. Activation of the HPG axis reaches a peak at six to eight weeks after delivery. In this period, the mini-puberty, the levels of sex steroids are similar to early-middle pubertal levels but their peripheral effects are not evident. Increased FSH in girls continues until the age of between two and four years, although estradiol is elevated from the second to the fourth month after delivery (1).

Premature thelarche (PT) refers to the precocious appearance of breast development in girls with no other signs of sexual maturation. It is mostly encountered during the first two years of life. PT has been postulated to result from transient, partial activation of the HPG axis with excessive secretion of FSH. The physiologic baseline event in PT is the increase in FSH level (2).

In females, antimüllerian hormone (AMH) is produced by the granulosa cells of primary, preantral and early antral follicles (3). AMH has at least two functions during follicular development. First, AMH plays an inhibitory role during initial recruitment, when resting primordial follicles are initiated to grow, and second, it may modify preantral and small antral follicle growth by decreasing the FSH responsiveness of the follicle. The second effect is important during cyclic recruitment, when some large preantral and small antral follicles are recruited to grow on to the preovulatory follicle stage (4,5,6).

AMH levels increase during infancy, but remain stable from childhood to early adulthood (7,8). In a recent study, it was reported that, after mini-puberty, AMH concentrations decreased by 30% during the first two years of life (9). It has also been reported that AMH concentrations in patients with central precocious puberty (CPP) were lower than those in the PT group and that there was a negative correlation between AMH and basal gonadotropin levels (10). These findings support the view that AMH may play a role in suppressing puberty. High concentrations of AMH in the mini-puberty period, when the peripheral effects of hormones are not usually observed despite the presence of hormonal values similar to those found during the pubertal period, supports the view that AMH may have a suppressive effect.

The aim of this study was to investigate AMH levels in infants with PT who are presumed to have a prolonged mini-puberty due to inadequate and/or late suppression of pubertal activation. We hypothesized that, the AMH mediated ovarian response which may prevent FSH-induced follicle growth is deficient in infants with PT.

## Methods

Forty five consecutive girls, aged between one and three years, who had been admitted to our hospital between July 2015 and September 2016 and who had PT, defined as breast development with no other signs of sexual maturation or bone age (BA) advancement, were included in the study. All parents received oral and written information before signing a consent form. The study was approved by the Local Ethical Committee (Zekai Tahir Burak Training and Research Hospital, no: 44/2015). Exclusion criteria were: central and peripheral precocious puberty; thyroid disorder; intake of any medication; acute or chronic disease; and small for gestational age.

Pubertal staging was performed according to the method of Marshall and Tanner (11) by the same pediatric endocrinologist in all subjects. If breast stages differed between the two breasts, the more advanced stage was assumed to represent breast development stage. All patients presented with breast budding as the only sign of puberty. Standard deviation (SD) scores (SDS) for height, weight and relative weight were calculated using national reference data (12). BA was evaluated using the Greulich and Pyle method by the same endocrinologist (13).

In all subjects, blood sampling was performed at 8:00 am, via an intravenous cannula inserted into an antecubital vein. The blood samples were drawn into standard vacuum tubes. For the AMH assay, samples were centrifuged (3000 × g for 10 min) within 30 minutes of drawing and serum was analysed immediately. AMH was measured by an enzyme immunoassay method (Anshlab AMH/MIS ELISA kit, Webster, Texas, USA). Luteinizing hormone (LH), FSH and estradiol were measured using a chemiluminescence method (Advia Centaur XP, Siemens AG, Munich, Germany).

All patients were prospectively examined by pelvic ultrasonography (US) performed by the same experienced pediatric radiologist, who was blinded to their clinical and laboratory findings. US was performed using a Logiq 6 US scanner (General Electric Co. Milwaukee, WI, USA) and a 7.5-MHz linear-array small parts transducer. Patients and controls were scanned several times until images with a full bladder could be captured. Foley catheterization was not performed in any of the participants. The three dimensions of the uterus, endometrial thickness, and the three dimensions of each ovary were measured. The fundocervical ratio was assessed as >1 or ≤1 as a simple and fast expression of uterine maturation. Endometrial echogenicity was checked with the uterus scanned in the sagittal plane. Ovarian volume, as cubic centimeters, was calculated using the ellipsoid formula (longitudinal dimension × AP dimension × transverse dimension × 0.52).

As the control group, 37 healthy, age-matched Tanner stage 1 infants were included in the study. SDS for height, weight and relative weight were calculated and AMH concentrations were measured in the control group.

### Statistical Analysis

The results of tests were expressed as the number of observations (n), mean ± SD, median and range or median and interquartile range as appropriate. The results of the homogeneity (Levene’s test) and normality tests (Shapiro-Wilk) were used to decide which statistical methods to apply in the comparison of the study groups. According to these test results, parametric test assumptions were not suitable for variables, so the comparisons of two independent groups were performed by using Mann-Whitney U test. A statistical significance level of p<0.05 was considered significant. Pearson’s correlation coefficient test was used for data with parametric test preconditions to determine the relationship between two continuous variables. In parametric test for variables that do not meet the pre-conditions, the Spearman correlation coefficient was used. All statistical analyses were performed using the Statistical Package for Social Sciences (SPSS) software, version 17 (IBM Inc., Chicago, IL, USA).

## Results

The anthropometric data of the patient and the control groups are given in Table 1. The mean±SD chronologic age (CA) of the patients was 1.76±0.54 (median 1.70; range 1-3) years. The mean±SD age of onset of breast development was 8.19±6.76 (median 8; range 0.8-22) months. The mean±SD duration of breast budding was 12.39±10.29 (median 10; range 1-34) months and the patient’s breast stages ranged from stage 2 to stage 3. The mean±SD CA and BA difference was 0.1±0.34 years (median 0.15; range -0.6 to+0.7). In the follow-up period (median 9 months; range 6-18 months), no pubertal progression was found in any patient. Serum AMH concentrations of PT subjects [median 1.66 ng/mL (11.85 pmol/L); range 0.15-6.32 ng/mL (1.07-45.12 pmol/L)] were significantly lower (p=0.025) than those of the control group [median 1.96 ng/mL (13.99 pmol/L); range 0.60-8.49 ng/mL (4.28-60.64 pmol/L)] (Table 1). When the patients were grouped by breast stage, there was no significant difference between their AMH levels (p=0.585). AMH was not correlated with CA and BA. There was no relationship between AMH and CA-BA difference.

**Figure 1:**
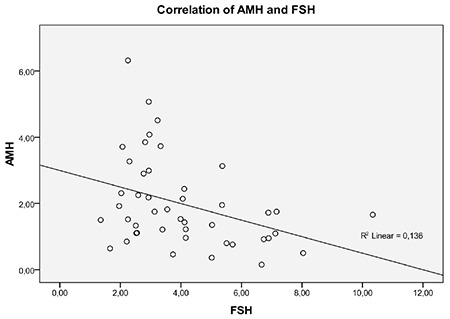
Correlation between antimüllerian hormone and follicle-stimulating hormone levels AMH: antimüllerian hormone, FSH: follicle-stimulating hormone

Laboratory and pelvic US findings of the infants with PT are given in Table 2. AMH and FSH were negatively correlated (r=-0.360; p=0.015) in infants with PT (Figure 1). The correlation between FSH and AMH levels was significant after controlling for the effect of age (r=-0.366; p=0.014). No correlation was found between AMH and LH and only four (7.3%) patients had a baseline LH concentration above the measurement limit, as expected. No correlation was found between AMH and estradiol concentration because estradiol concentrations were above the measurement limit in only 16 (35.5%) patients. No correlation was found between AMH and LH concentrations. Uterine length was greater than 34 mm in only two patients and uterine volume was above 2 mL in five patients. Endometrial echo was detected in four patients. There were six patients with a fundocervical ratio above one. There was no correlation between AMH levels and uterine size, uterine volume, endometrial thickness, fundocervical ratio, ovarian size or volume, follicle size and follicle number.

## Discussion

This is the first study investigating AMH concentrations in infants with PT. In this study, serum AMH levels in infants with PT were significantly lower than those of healthy girls and a negative correlation between FSH and AMH was detected. Evidence in the literature suggests that AMH concentrations increase gradually from three years before pubertal onset to the beginning of puberty and then decrease by approximately 30% during the following two years (9,14,15). In a recent study, it was reported that the AMH levels of patients with CPP were lower than AMH concentrations in PT patients, aged between 4.5-8 years and that a negative correlation existed between AMH and basal and stimulated gonadotropin levels (10). AMH plays an inhibitory role during initial recruitment, when resting primordial follicles are initiated to grow and at the same time AMH decreases the sensitivity of primordial follicles to FSH and inhibits granulosa cell aromatase, which results in a decreased chance of the follicle moving towards cyclic recruitment and estrogen biosynthesis (4,5,6). The decrease in AMH concentration during the activation of the hypothalamus-pituitary-ovarian axis causes cyclic follicle recruitment. Partial activation of the hypothalamus-pituitary-ovarian axis, during so-called mini puberty, in the first months of life in girls has been demonstrated. An increase in AMH concentrations from birth to three months of age has also been reported (7). As AMH inhibits both initial follicle recruitment (primordial to primary follicles) and FSH-dependent follicular growth (preantral and antral follicles), these authors suggested that rising levels of AMH during mini puberty may be an ovarian response to prevent FSH-induced follicular growth at a time of life when further differentiation of follicles would be inappropriate. Elevated AMH concentrations might prevent the progress of puberty.

PT is a condition seen during the period of mini-puberty and it has been postulated to result from prolonged mini-puberty due to inadequate and/or late suppression of pubertal activation (1,2). In this present study, detection of somewhat lower AMH concentrations in infants with PT and a negative correlation between FSH and AMH are findings which support the hypothesis that an AMH-related ovarian response which inhibits FSH-induced follicular growth is deficient in infants with PT (power of test=45%). However, the role of AMH in the pathogenesis of PT is not well clarified. It is known that many factors have complex interactions during the mini-puberty period. The findings of this present study support our proposition that AMH may play a role in this complex interaction.

Results reported from five other studies investigating AMH in early pubertal development are controversial (Table 3) (10,16,17,18,19). In one study, the number of subjects was very limited and no comparison was made with healthy controls (16). In a second study, the groups consisted of girls of ages 4-8 years and the AMH concentrations in patients with CPP were found to be lower than those of the PT group; results consistent with the results of this present study (10). In another study, compared with slowly progressive CPP, girls with more rapidly progressive CPP were reported to have lower AMH concentrations (18). This result also supports the proposition that pubertal progression is associated with decreased AMH concentrations. In another study, however, no difference in AMH concentrations was reported between CPP patients and the control group. However, in this study the patients were older, with more advanced pubertal stages (17). Thus, it was not possible to determine if the decrease in AMH reported at the onset of puberty was due to advanced pubertal stage. In another recent study, serum AMH concentrations in girls with PT were found to be higher than those of prepubertal girls. However, in this study the age of the control group was significantly different to that of the PT group, making a comparison inappropriate (19).

Pelvic ultrasound might be useful for diagnosis of precocious puberty. However no significant differences in uterine and ovarian ultrasound measurements were detected between children with PT and controls (2). In a recent study, AMH was reported to be proportional to the number of small (2-3 mm) and medium (4-6 mm) follicles. Thus, in early puberty (Tanner breast stage 1-3), the number of AMH-producing follicles (2-6 mm) correlated positively with pubertal stages, whereas AMH levels were unaffected (20). In our study, ultrasound findings being prepubertal in the great majority of our patients, as expected, we were not able to determine a correlation between AMH and pelvic ultrasound findings.

### Study Limitations

The study and control groups were composed of infants. Thus, due to ethical reasons, only AMH concentrations were measured in the control group. Although a negative correlation between AMH and FSH was found in the PT group, the relationship between FSH and AMH in the controls would have allowed comparison with the PT group if FSH concentrations had also been available from the control group.

## Conclusion

In conclusion, AMH may play a role in suppressing pubertal findings during infancy. This effect of AMH might be due to the decrease in the sensitivity of primordial follicles to FSH and inhibition of granulosa cell aromatase which results in a decreased chance for the follicle to move toward cyclic recruitment and estrogen biosynthesis. Decreased AMH may cause PT in infants. In this present study, AMH concentrations in infants with PT were significantly lower than those found in healthy controls of the same age. A negative correlation was also found between AMH and FSH. Although our findings support this hypothesis, the opposite hypothesis, namely, that an excessive activation of the ovary results in lower AMH production cannot be completely excluded and the influence of other factors involved in mini-puberty cannot be ruled out. The cause of somewhat lower AMH concentrations and the role of AMH in the etiopathogenesis of PT should be clarified by further studies evaluating AMH levels in mini-puberty and related disorders in infancy.

## Figures and Tables

**Table 1:**
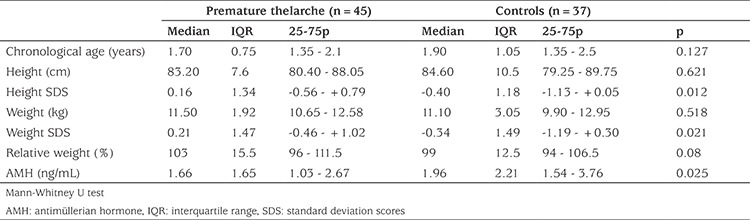
Anthropometric properties of infants with premature thelarche and the control group

**Table 2:**
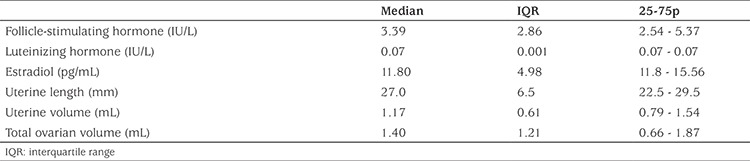
Laboratory and ultrasound findings of infants with premature thelarche

**Table 3:**
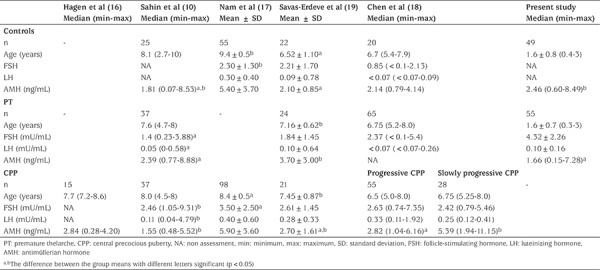
Reported antimüllerian hormone levels in early pubertal development
